# Using Open Source Data to Identify Transit Deserts in Four Major Chinese Cities

**DOI:** 10.3390/ijgi9020100

**Published:** 2020-02-06

**Authors:** Junfeng Jiao, Mingming Cai

**Affiliations:** 1 Urban Information Lab, the University of Texas at Austin, Austin, TX 78705, USA; 2 School of Resource and Environmental Sciences, Wuhan University, Wuhan 430079, China

**Keywords:** transit desert, transit demand, transit supply, China

## Abstract

The concept of transit deserts stems from the concept of food deserts. There is substantial research on transit deserts in developed countries. However, there is no known research that has studied this subject in Chinese cities. Using open-source data, this paper identified transit desert areas in four major Chinese cities (Beijing, Shanghai, Wuhan, Chengdu). The results show that: (1) In these four cities, the transit desert areas are mainly concentrated in city centers and hardly occur in any suburban areas, which is very different from the cases in the US. (2) Shanghai has the largest transit-dependent population living in transit deserts, followed by Beijing, Chengdu, and Wuhan. Chengdu has the smallest transit desert areas, followed by Shanghai, Wuhan, and Beijing. (3) An oversized transit-dependent population and incomplete transit systems in these cities might contribute to the transit deserts’ occurrences. (4) Different distribution of population density, traveling preference, and transportation investment policy in Chinese and American cities might contribute to the different findings. By examining transit desert problems in major Chinese cities, this study brought people’s attention to the gap between transit demand and supply in China.

## 1. Introduction

The concept of “transit deserts” originates from the popular and thoroughly studied concept, “food deserts,” which refers to those areas lacking enough access to fresh and nutritious food for residents [1,2]. Similarly, a transit desert is defined as an area that is not equipped with enough transit services to meet residents’ travel demands [3]. It reveals that the level of transportation accessibility in this area is low, caused by deficiencies in transport infrastructure and connecting services, or the transit-dependent population is overcrowded [4]. It will take people a far longer time to travel through than the other areas. People residing in the transit desert areas suffer from the low level of transit access or fiercer competition for transit services. The identification of transit deserts involves three major concepts. The first is transit demand. It refers to the demand for transit services of residents who live in an area, measured by the total number of the transit-dependent population in this area. Transit-dependent populations are likely to require public transit services to travel within urban areas compared to other populations. This group often includes juveniles, the elderly, those lacking the financial capacity for private vehicles, or those who are unable to drive [5]. The second is transit supply, which shows the conditions of transit services spatially connecting different areas and carrying human mobility in the area. It is measured from three aspects: transit stops, transit routes, and road networks. Lastly, the transit gap, which can be measured by subtracting demand from supply. The areas with high transit gaps, where transit demands are far beyond transit supplies, are identified as transit deserts.

Since 2013, transit desert research has undergone comprehensive development in the US and been applied to many cities. Studies showed that most transit deserts occurred in neighborhoods surrounding central urban historic areas, whereas a smaller number of transit deserts was scattered in isolated suburbs of major American cities [3,6]. Measurements for transit demand and supply were further refined as well, by adding the number of transit stops, routes, and trips, the length of low-speed roads, and the density of road intersections [6]. Previous studies show that in most American cities, transit services are likely concentrated in the city center. Conversely, transit demand tends to be scattered around the entire city [7]. Recently, transit deserts have been used as a tool to identify existing housing and employment problems in American cities. For example, research showed that over 400,000 residents and 250,000 job opportunities are located in transit deserts in Cook County, Illinois [8]. On average 4 million or 7% of the population of the 52 major American cities live in transit deserts [9].

There is little research on this subject for Chinese cities. China is a developing country which has gone through a rapid socioeconomic development and urbanization process in the past forty years [10-12]. Transportation infrastructure in Chinese megacities developed quickly, expanding from central urban areas to suburbs, while promoting inter-city and intra-city transportation [13,14]. Due to this rapid urbanization, most cities’ transportation systems do not meet the transportation demand. Additionally, there are problems associated with non-motorized travel in Chinese cities. In recent years, walkability in Chinese cities has gradually decreased, especially in newly developed suburbs [15,16]. Bike lane density in Chinese cities also fell significantly [17]. According to a 2018 report on urban commuting in China released by Jiguang Company, Chinese urban residents suffer from high transit service inequality, partly due to an oversized population and imperfect transit facilities [18]. Clearly, solutions for alleviating transit inequalities in overpopulated cities is an emergent issue. Urban planners and policymakers continue to implement policies to solve this problem, but more comprehensive studies should be conducted to understand the current state of public transit in China. Therefore, identifying transit deserts in major cities in China is an important question for both academic research and planning practice.

Using open-source data, this study attempts to fill the research gap by investigating the relationship between transit supply and demand in four major Chinese cities (Beijing, Shanghai, Wuhan, and Chengdu) which are located in the east, the middle, and the west of China, respectively. This study intends to address three primary questions: (1) What are the characteristics of the areas with large transit gaps between supply and demand in major Chinese cities? (2) What are the similarities and differences between the spatial distributions of these areas in Chinese and American cities? (3) What might cause these differences?

## 2. Methods and Materials

### 2.1. Study Area

**Figure 1.**
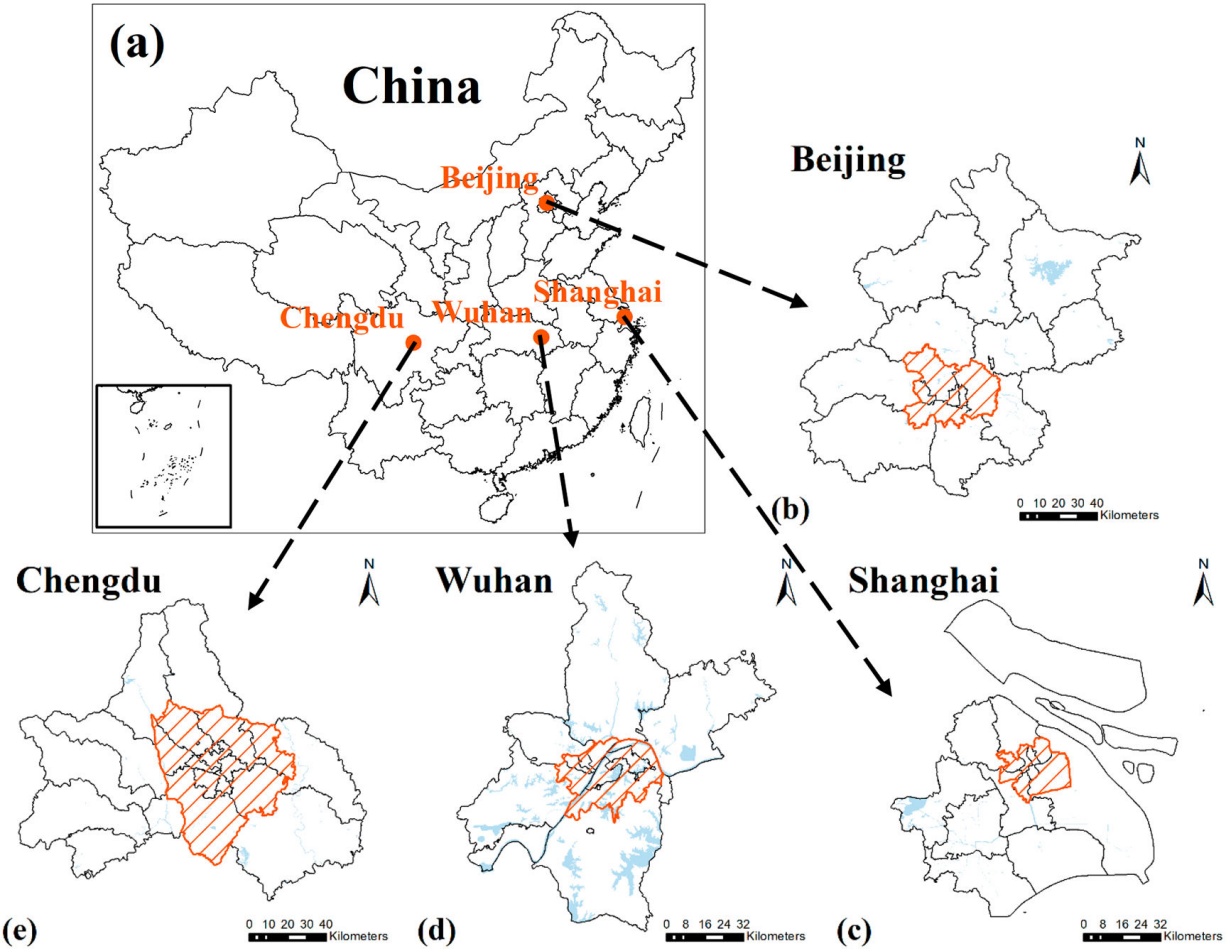
The study areas: (**a**) map of China; (**b**) map of Beijing, China; (**c**) map of Shanghai, China; (**d**) map of Wuhan, China; (**e**) map of Chengdu, China. The shaded area refers to the central urban areas of each city.

This study focuses on four major Chinese cities: Beijing, Shanghai, Wuhan, and Chengdu (Figure 1). These four cities are major political and economic centers in China with large populations and well-developed transportation systems. They are located in different geographical regions of China. Beijing and Shanghai sit in the northeast, with Beijing more inland than Shanghai. Wuhan, known as “the River City” and “the City of Hundreds of Lakes,” is located in the middle of the country. Chengdu is the central city of western China and plays an important role in China’s western development program.

### 2.2. Data

All the data used in the research were collected from open sources. Road network data was collected from the OpenStreetMap which is crowdsourced and provides free geographic data to the world. The data consists of various kinds of road layers, such as motorways, major, pedestrian streets, and cycleways. Among these layers, footways, living streets, and pedestrian lanes are identified as sidewalks, while cycleways are identified as bike routes. Sidewalks, bike routes, and residential streets are extracted as low-speed roads. Data for transit stops and routes were collected from Amap.com via calling the resources through an application programming interface (API) of the website. Amap is a open platform belonging to Amap Company that creates map products and provides map services to users. By sending requests to a uniform resource locator (URL) connected with a certain map service, the information recorded in the URL is sent back via its network port. The transit trips are collected from public transit timetables, which include start time, end time, and interval time. Population data were collected from a 2017 LandScan dataset of Oak Ridge National Laboratory (ORNL) and the 2010 Chinese Sixth Demographic Census. The ORNL’s LandScan datasets provide global population distribution data based on a 30 m × 30 m spatial resolution [19].

Since LandScan population added the number of permanent residents and migrant population in China together, we modified the LandScan population number by the total permanent population number recorded in the 2018 Chinese Statistical Yearbook which provides the statistics data of China in 2017 [20]. Due to the lack of age data in 2017, the researchers applied the age data of the 2010 Sixth Demographic Census to calculate the transit-dependent population. In addition, the number of private vehicles was collected from data at the city level published by the cities’ transport bureaus and transport industry reports from associated transit agencies. The unit of analysis is subdistrict, which on average is 51 km^2^ and includes 85,000 people. Finally, all data were transformed from WGS84 geographical coordinate system to the UTM (UniversalTransverseMercator) projection coordinate system.

### 2.3. Methodology

A negative transit gap value means transit supply is less than demand in the area, while a positive value means transit supply exceeds demand [21]. A large negative gap shows that transit supply is not enough to satisfy demand in the area. The lower the value, the larger the gap between transit demand and supply. Conversely, the greater the value, the smaller the gap. The areas with large negative transit gaps are identified as transit desert areas.

The transit desert analysis was conducted in four Chinese major cities (Beijing, Shanghai, Wuhan, and Chengdu) at the subdistrict level. A subdistrict is an administrative unit in Chinese cities which is one level lower than a district. The area of a subdistrict is close to the size of a town in American cities. For transit-dependent population, the research used a formula developed by the US Department of Transportation to measure the transit-dependent populations within each subdistrict of each city [22]. First, residents aged 16 years or over were considered a population that might be drivers. Then, the number of drivers was calculated by subtracting the number of people residing in group quarters (the residences that companies or institutes provide to their employees) from the population aged 16 and above. The transit-dependent population was measured by subtracting the number of private vehicles from household drivers (Equation (1)). After the transit-dependent population was calculated for each subdistrict, the value was then divided by the area of the subdistrict to calculate the density of transit-dependent populations. Finally, the Z-scores were measured for the density of transit-dependent populations in each subdistrict for normalization (Equation (2)).

(1) $$TDP_{i}=(TP_{i}*EP_{i}-QP_{i}-AV_{i})∕S_{i}$$

(2) $$TD_{i}=\frac{TDP_{i}-Aver(TDP_{i})}{STD(TDP_{i})}$$

where $TDP_{i}$ refers to the density of transit-dependent population in subdistrict i; $TP_{i}$ refers to the total population number in 2017 in subdistrict i; $EP_{i}$ is the proportion of people aged 16 and above in 2010 in subdistrict i; $QP_{i}$ is the number of persons living in group quarters in subdistrict i; $AV_{i}$ is the number of private vehicles in subdistrict i; and $S_{i}$ is the area of subdistrict i. $TD_{i}$ refers to the transit demand in subdistrict i, presented by the Z-scores of the density of transit-dependent population. $TDP_{i}$ is the density of transit-dependent population in subdistrict i, while $Aver(TDP_{i})$ and $STD(TDP_{i})$ are the arithmetic mean and the standard deviation of all density values, respectively.

People living in group quarters were identified by extracting population grids where there are points of group quarters. Then, the number of people residing in group quarters of each subdistrict was measured by the total population in the grids. Specifically, group quarters include such places as college residence halls, residential treatment centers, skilled nursing facilities, group homes, military barracks, correctional facilities, and workers’ dormitories, which is the same as the definition of group quarters according to the US Census. At the same time, data for private vehicles are not published at the subdistricts’ level. Thus, we calculated the number of private vehicles in a subdistrict by multiplying total private vehicles in the entire city with the ratio of total population in this subdistrict to total population in the city and the ratio of gross domestic product (GDP) share in this subdistrict to total GDP in the city. If a subdistrict has more private vehicles than residents, its transit-dependent population would be adjusted to zero.

Transit supply in subdistricts of each city was calculated by aggregating the following seven criteria including the numbers of transit stops, routes, and trips, the total lengths of sidewalks, bike routes, and low-speed limit roads, and the number of road intersections [23,24]. The number of transit stops, routes, trips, and road intersections reveals the services of motorized transit systems, while the lengths of sidewalks, bike routes, and low-speed limit roads are used to evaluate the non-motorized (walking and cycling) and low-speed transit systems. Since motorized and non-motorized transit systems jointly constitute to urban transportation systems, we weighted these seven variables equally to measure the conditions of transit services supply in an area. Transit trips represent the frequency of transit services within an area, which are collected based on weekday services. However, not all transit routes have complete information to calculate trips. For those routes including only a start and end time, the research set the interval time as 15 min. For the routes only including start time, the end time was set to 22:30, and the interval time was also set to 15 min. Once these values were calculated for each subdistrict, they were divided by the area to calculate the density of transit supply of each subdistrict. Z-scores were calculated and aggregated for these seven criteria to represent the overall transit supply of the subdistrict.

(3) $$TS_{i}=(\text{TSZ}+\text{TRZ}+\text{TTZ}+\text{SLZ}+\text{BLZ}+\text{LLZ}+\text{RIZ})∕7$$

where $TS_{i}$ is the transit supply in subdistrict i; TSZ, TRZ, and TTZ are the Z-scores of the density of transit stops, routes, and trips within subdistrict i; SLZ, BLZ, and LLZ are the Z-scores of the density of sidewalks, bike routes, and low-speed limit roads, respectively; IDZ refers to the Z-score of the density of road intersections in subdistrict i.

Transit gaps for each subdistrict were calculated by subtracting the Z-scores of transit demand from those of supply. Finally, five levels of transit gaps were classified by the natural breaks method, and the areas with the highest and second highest levels of transit gaps were identified as the transit desert areas.

(4) $$TG_{i}=TS_{i}-TD_{i}$$

where $TG_{i}$ refers to the transit gap in subdistrict i.

## 3. Results

The characteristics of transit deserts in the study areas are analyzed from three aspects: transit demand population, transit system, and the gaps between demand and supply. For these four cities, Shanghai has the highest proportion of transit-dependent populations (14.5 million, 62.01%), followed by Beijing (11 million, 50.51%). Wuhan and Chengdu have relatively lower percentages of transit-dependent populations; they are 2.3 million or 21.16% and 5 million or 31.22%, respectively (Table 1). All these cities have more than 20% transit-dependent population which is different from cities studied in Texas, of which the case studies used the identical methodology with this study [6]. Shanghai has the largest number of transit-dependent people living in transit deserts (2,836,960), followed by Beijing (1,721,971). Chengdu (347,032) and Wuhan (50,037) have transit-dependent populations living in transit deserts that are smaller than those in Shanghai and Beijing. Chengdu has the smallest transit desert areas (36.80 km^2^), followed by Shanghai (57.38 km^2^), Wuhan (61.49 km^2^), and Beijing (109.94 km^2^).

In terms of the transit supply, Beijing and Shanghai operate much larger transit systems than Wuhan and Chengdu, even though Shanghai has the smallest geographic area among these four cities. Beijing operates the largest transit system in comparison to the other cities. Beijing’s transit system covers 16,360 km^2^ and includes 10 rail lines, 1658 bus lines, and 14,640 bus stops. Beijing also has far longer sidewalks (1398.09 km) and more low-speed roads (6683.32 km) than the other cities. Shanghai has the most rail lines (17 lines), the highest frequency of transit service (681,452 trips), and the longest bike routes (414.30 km) among the studied cities. Due to its oversized population crowded into a relatively small urban area, Shanghai has a much denser road network and road intersections (18.90 per km^2^) than the other three cities. Shanghai also has 15 boat lines, which is unique to Shanghai. Wuhan has the fewest transit stops (6102), bus routes (656), sidewalks (260 km), and low-speed roads (2200 km) among these cities. However, it does have more subway lines (12) and a more frequent transit system (332,435 trips per day) than Chengdu (281,610 trips). Chengdu’s geographical areas are comparable to Beijing but with a much smaller transit system coverage and only concentrated in central urban areas, which results in the lowest road intersection density among the cities (3.75 per km^2^). Chengdu also has the fewest subway lines (4 lines), the shortest bike routes (47.67 km), and the lowest frequency of transit services (281,610 trips per day), although it does have a very large transit-dependent population.

**Figure 2.**
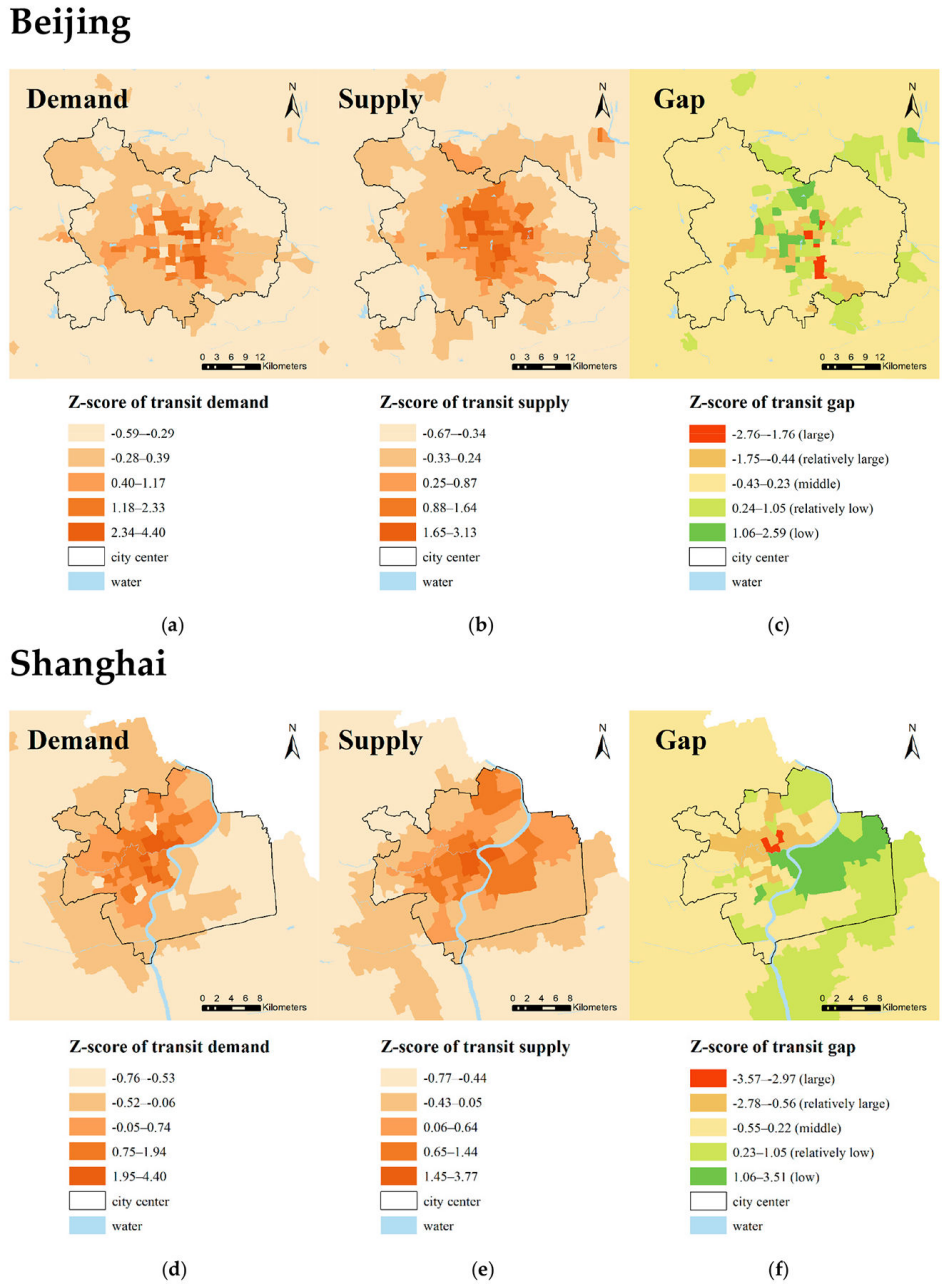
Transit gaps between supplies and demands in Beijing and Shanghai: (**a**) transit demands in Beijing; (**b**) transit supplies in Beijing; (**c**) transit gaps in Beijing; (**d**) transit demands in Shanghai; (**e**) transit supplies in Shanghai; (**f**) transit gaps in Shanghai.

**Figure 3.**
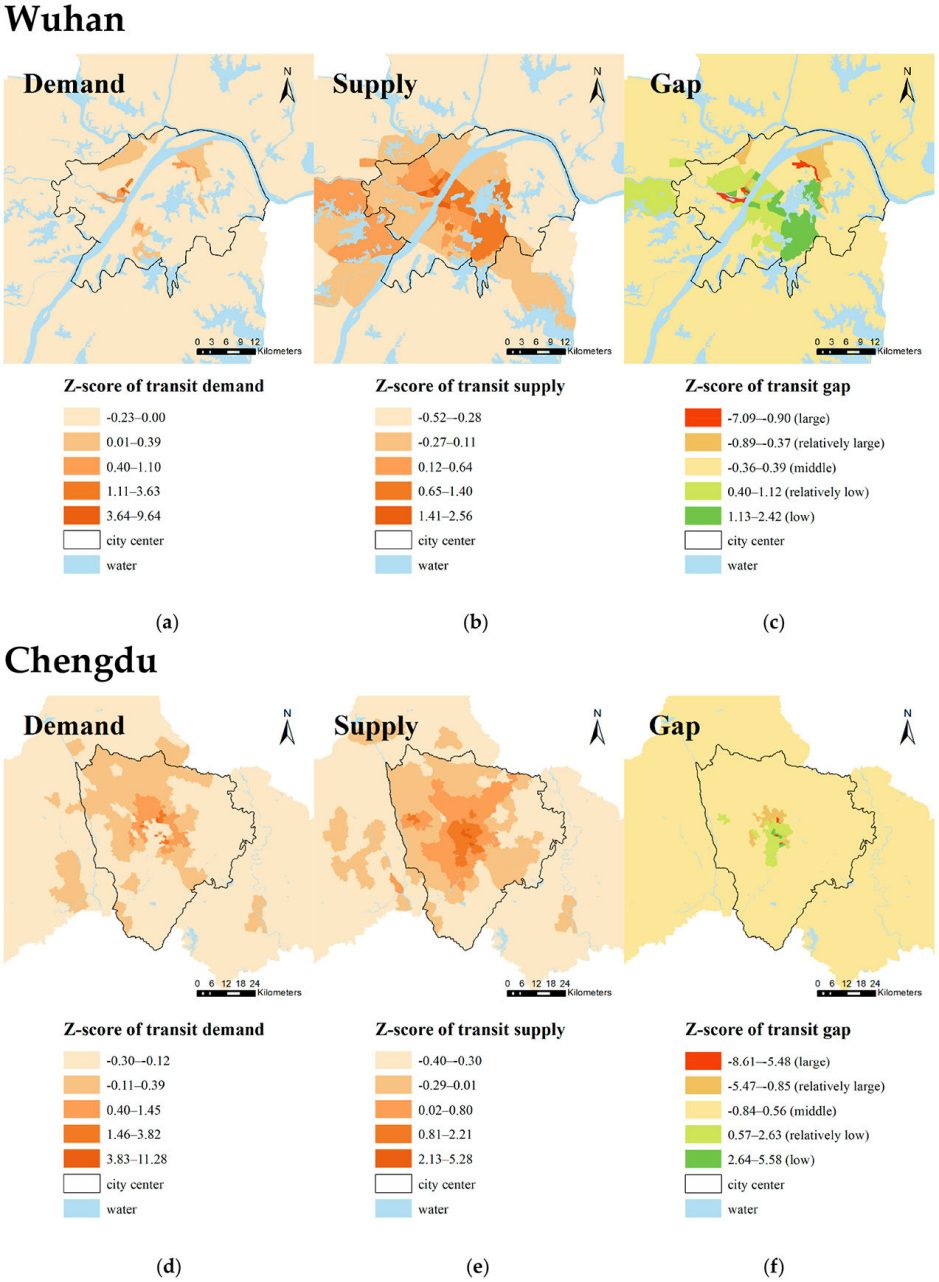
Transit gaps between supplies and demands in Wuhan and Chengdu: (**a**) transit demands in Wuhan; (**b**) transit supplies in Wuhan; (**c**) transit gaps in Wuhan; (**d**) transit demands in Chengdu; (**e**) transit supplies in Chengdu; (**f**) transit gaps in Chengdu.

Figures 2 and 3 show the spatial distribution of transit demand, transit supply, and transit gaps in these four cities. Table 2 shows the top five subdistricts with the largest and smallest gaps between transit supply and demand in each city. Overall, Chengdu has the smallest proportion of the areas where transit supply is unable to satisfy demand, followed by Wuhan, Shanghai, and Beijing. Spatially, the transit supply and demand of most subdistricts in each city are relatively balanced. The results show that in these four cities, the transit desert areas are concentrated in the central urban areas where the levels of transit demand are also very high. The results also show that most areas with enough transit supply for demand are concentrated in the city center, whereas some of them are scattered in the urban outskirts (e.g., Beijing).

## 4. Discussion

The research identified transit deserts in four major Chinese cities. Overall, most of these cities have a high concentration of transit desert areas in the central urban areas, especially in historic old towns. The spatial distribution characteristics of transit demand and supply in Chinese metropolitan cities are significantly different from those in American cities, especially in suburbs. In suburban areas, areas where transit supply cannot meet demand hardly occurs, which differs from the cases in American cities where transit deserts are scattered in isolated suburbs [5,7]. For these four cities, Chengdu and Beijing respectively have the smallest and largest transit desert areas. Shanghai has the highest proportion of transit-dependent populations, followed by Beijing, Chengdu, and Wuhan.

For transit supply, transit services are mainly concentrated in the city center of all four cities. The density of transit service decreases as distance from the city center increases, which confirms previous studies [8]. Likewise, in terms of transit demand, areas with higher proportions of transit-dependent populations are concentrated in city centers. However, areas with denser transit service spread more widely than those areas with high transit demand in each city. In Beijing and Chengdu, some areas with a higher level of transit services supplies are separated from the other areas, while in Shanghai and Wuhan, the areas with more transit supplies are clustered together.

There are various causes of transit deserts in these four cities. In Beijing, the subdistricts around Dongzhimen in Dongcheng District are categorized as transit desert areas due to the transit demand far exceeding the transit supply. The high density of both middle schools and job opportunities might explain this finding. There are also transit deserts around Longtan Park in Dongcheng District with transit demand exceeding supply. It may be because of the lack of sidewalks and bike lanes. Like Beijing, Shanghai also has transit desert areas located around urban economic centers, along Baoshan Road across Jingan District and Hongkou District. Insufficient transit service supply might stem from a lack of sidewalks and bike routes. Wuhan has more transit deserts compared to the other cities. In Wuhan, several districts have poor public transit services for transit demand, especially in the south of Jiangxia District. Some of the largest transit gaps in Wuhan are distributed in Jianghan District. Although the area has a concentrated distribution of transit stops and high transit service frequency, transit demand still far exceeds supply owing to large population density, which results in major transit deserts in Wuhan. Dissimilar to the other three cities, transit deserts in Chengdu are mainly a result of the oversized transit-dependent population and inadequate non-motorized roads in historic downtowns. To solve these problems, the city of Chengdu government might consider significantly increasing transportation investment in these areas.

The differences between transit deserts in China and the US might be a result of the distribution of population density, traveling preference, and transportation investment policies. High population density promotes the development of public transportation in Chinese cities. The residents in these four cities rely more on the public transit systems than the people in American cities. Unlike China, most people in American cities have a preference to commute by private automobiles due to the sprawling development patterns [25-27]. Moreover, in recent years, increasing investments in highways and major expressways conducive to facilitating agglomeration economies along corridors connecting nodes has contributed to fewer resources being spent on public transit between the areas with low population density where low-income families mainly resided [28,29]. In view of fiscal costs and profits, transit systems often only operate around downtown and connect outskirts to the city center where the majority of people travel between. Therefore, transit supply has a higher likelihood of satisfying demand in downtown areas of American cities, with transit deserts often found in newly developed suburban areas. This is very different from Chinese cities. Due to the rapid urbanization in the past several decades, the population has sharply expanded across the entire urban area. Urban transit systems in these four major Chinese cities have undergone rapid development in accordance with dispersed populations [14]. Transit systems have expanded to cover both the city center and most suburban areas, providing residents in various areas with more mobility options and better transit accessibility. People are prone to depend on public transportation because of its cost compared to personal automobile usage as well as its convenience and safety. It seems that transportation equity in Chinese cities is better than in American cities. However, since a large amount of the population in Chinese cities lives inside or nearby downtown areas, high transit demand has created many transit deserts near downtown. Transit deserts in both American and Chinese cities do share some common characteristics. For example, an oversized population and old transit system in central historic downtowns often create transit deserts. Therefore, to remove transit deserts, it is necessary to improve transportation investments in these areas, requiring joint efforts from both local governments and public transportation operators.

The research has some limitations. The authors mainly used open-source data from different sources to calculate transit demand and supply, which may create unintentional errors. Official data from governments might solve this problem for future research. Further, the methodology used in this study did not consider the interaction between the built environment and transit services supply, such as the space quality near transit systems, traffic congestion, and vehicle exhaust, which may have significant effects on people’s feelings and health. The next step of the research could investigate the relationship between transit systems and other urban land uses, considering transportation equity and sustainability.

## 5. Conclusions

This paper demonstrates a quantitative method to identify transit demand, transit supply, and transit gaps in four major Chinese cities. The results show that transit deserts in Chinese cities are mainly concentrated in the city center, which is very different from cases in American cities where transit deserts are scattered in isolated suburbs. Shanghai has the largest transit-dependent population living in transit deserts (almost 8 million), followed by Beijing, Chengdu, and Wuhan. Chengdu has the smallest transit desert area (about 37 km^2^), followed by Shanghai, Wuhan, and Beijing. As for the causes of transit deserts, there is no totally identical reason that applies to all four cities. However, either the oversized transit-dependent populations or insufficient transit services supply contribute to the occurance of transit deserts. The differences between transit deserts in Chinese and American cities could be explained by the varying distribution of population density, traveling preference, and transportation investment policy. Most people in American cities rely on private automobiles for mobility demand. Governments only provide enough transit service for people around downtown and corridors between downtown areas and suburban areas. Therefore, transit deserts often occur in newly developed suburbs or historic downtowns with relatively poor public transit. Conversely, residents of large Chinese cities rely on public transit for mobility demand. Local governments have developed extensive and increasingly mature public transit even in suburban areas. Thus, suburban Chinese residents may have better accessibility via public transit than residents in American cities. However, due to the oversized transit demands within urban centers or the regions adjacent to, but out of, urban centers, we often find transit deserts in these areas. This research introduces the transit deserts concept to China and helps us further understand the state of transit supply, demand, and gaps in major Chinese cities.

## Figures and Tables

**Table 1.** Characteristics of transit and built environment in Chinese metropolitan cities.

Criteria	Beijing	Shanghai	Wuhan	Chengdu
Population	21,707,000	23,480,000	10,892,900	16,044,700
Transit Dependent Population (TDP)	10,964,631	14,560,829	2,305,414	5,008,891
Proportion of TDP	50.51%	62.01%	21.16%	31.22%
Size of Transit Deserts (km^2^)	109.94	57.38	61.49	36.80
Percentage of Transit Desert Areas	0.67%	1.29%	0.72%	0.26%
TDP Live in Transit Deserts	1,721,971	2,836,960	50,037	347,032
Number of Transit Smiddles	14,640	12,557	6102	9295
Density of Transit Smiddles (numbers/km^2^)	0.89	2.83	0.71	0.65
Number of Transit Routes	1658 bus lines 10 subway lines	1313 bus lines 15 boat lines 17 subway lines	656 bus lines 12 subway lines	734 bus lines 4 subway lines
Density of Transit Routes (numbers/km^2^)	0.10 bus lines 0.0006 subway lines	0.30 bus lines 0.0033 boat lines 0.0038 subway lines	0.08 bus lines 0.0014 subway lines	0.05 bus lines 0.0003 subway lines
Number of Transit Trips (24 h, weekday)	456,783	681,452	332,435	281,610
Density of Transit Trips (numbers/sq.km.)	27.92	153.64	38.95	19.75
Length of Sidewalks (km)	1398.09	753.76	260.01	475.31
Density of Sidewalks (km/sq.km.)	0.086	0.170	0.031	0.033
Length of Bike Routes (km)	391.57	414.30	148.14	47.67
Density of Bike Routes (km/sq.km.)	0.024	0.093	0.017	0.003
Length of Low-Speed Roads (km)	6683.32	4020.01	2200.41	3501.00
Density of Low-Speed Roads (km/km^2^)	0.41	0.91	0.26	0.25
Intersections	104,788	83,822	32,278	53,444
Density of Intersections (numbers/km^2^)	6.41	18.90	3.78	3.75
Urban Area (km^2^)	16,359.57	4435.43	8535.93	14,257.49

**Table 2.** Characteristics of transit gaps between supply and demand in Chinese metropolitan cities.

City	Largest Transit Gaps		Smallest Transit Gaps	
	District	Gap	District	Gap
	1. Xiangheyuan	−2.76	1. Zhanlan Road	2.59
	2. Jiaodaokou	−2.66	2. Zhongguancun	2.24
Beijing	3. Tiyuguan Road	−2.59	3. Chunshu	2.05
	4. Wanzhuang	−2.40	4. Dongsi	2.01
	5. Andingmen	−2.37	5. Baizhifang	1.78
	1. Tianmuxi Road	−3.57	1. Nanjingdong Road	3.51
	2. Sichuanbei Road	−3.25	2. Lujiaozui	1.91
Shanghai	3. Beizhan	−2.79	3. Yuyuan	1.90
	4. Dapuqiao	−1.89	4. The Bund	1.86
	5. Shimen Second Road	−1.69	5. Gold Yangxin Village	1.54
	1. Minyi Street	−7.09	1. Minzu Street	2.42
	2. Minquan Street	−1.19	2. Hualou Street	2.03
Wuhan	3. Changqian Street	−1.02	3. Ronghua Street	1.98
	4. Qingchuan Street	−0.99	4. Zhonghua Road	1.90
	5. Wugang Factory	−0.79	5. Liangdao Street	1.88
	1. Duyuan Street	−8.61	1. Yanshikou	5.58
	2. Qinglong Street	−5.95	2. Hejiangting	4.31
Chengdu	3. Longzhou Road	−5.48	3. Dongguang	3.60
	4. Shahe Street	−2.81	4. Wangjiaguai	3.33
	5. Shuanggui Road	−2.66	5. Shuijingfang	2.88
